# 5-hydroxymethylcytosine and its potential roles in development and cancer

**DOI:** 10.1186/1756-8935-6-10

**Published:** 2013-05-01

**Authors:** Gerd P Pfeifer, Swati Kadam, Seung-Gi Jin

**Affiliations:** 1 Department of Cancer Biology, Beckman Research Institute, City of Hope, 1500 East Duarte Road, Duarte, CA, 91010, USA

## Abstract

Only a few years ago it was demonstrated that mammalian DNA contains oxidized forms of 5-methylcytosine (5mC). The base 5-hydroxymethylcytosine (5hmC) is the most abundant of these oxidation products and is referred to as the sixth DNA base. 5hmC is produced from 5mC in an enzymatic pathway involving three 5mC oxidases, Ten-eleven translocation (TET)1, TET2, and TET3. The biological role of 5hmC is still unclear. Current models propose that 5hmC is an intermediate base in an active or passive DNA demethylation process that operates during important reprogramming phases of mammalian development. Tumors originating in various human tissues have strongly depleted levels of 5hmC. Apparently, 5hmC cannot be maintained in proliferating cells. Furthermore, mutations in the *TET2* gene are commonly observed in human myeloid malignancies. Since TET proteins and many lysine demethylases require 2-oxoglutarate as a cofactor, aberrations in cofactor biochemical pathways, including mutations in isocitrate dehydrogenase (*IDH*), may affect levels of 5hmC and 5mC in certain types of tumors, either directly or indirectly. We discuss current data and models of the function of 5hmC in general, with special emphasis on its role in mechanisms of development and cancer.

## Introduction

5-methylcytosine (5mC) is created in a postreplicative enzymatic reaction in which a DNA methyltransferase enzyme transfers a methyl group from S-adenosylmethionine onto the 5-carbon of cytosine, mostly within the CpG sequence context [1]. Presence of 5mC at gene promoters is most often linked to transcriptional repression [2]. It was long thought that 5mC was the only modified base in animal DNA. 5-hydroxymethylcytosine (5hmC) was initially found in the DNA of certain bacteriophages [3] and was reported in mammalian tissues as early as 1972 [4]. However, the levels reported by Penn *et al*. [4] seemed too high and could not be confirmed in subsequent studies [5]. The earlier report by Penn *et al*. [4] had put the levels of 5hmC in brain and liver DNA at 15% of the level of cytosine, which is at least an order of magnitude higher than currently established levels for brain and around two orders of magnitude higher than levels found in liver DNA [6]. Also, in the same study, 5mC was not detected casting doubt on these earlier results.

It was not until 2009 that the existence of 5hmC in mammalian cells was unambiguously proven [7,8]. By homology searches against a bacteriophage protein that oxidizes thymine in DNA, Tahiliani *et al*. [8] discovered three proteins, Ten-eleven translocation 1–3 (TET1-3), in mammalian genomes as candidate 5mC oxidases and confirmed such activity for TET1, a gene/protein earlier implicated in a translocation in a myeloid leukemia patient [9]. These discoveries were breakthroughs in the field of mammalian epigenetics.

## Review

### Dual role of 5-hydroxymethylcytosine as a stable DNA base and as an intermediate in DNA demethylation

**Figure 1:**
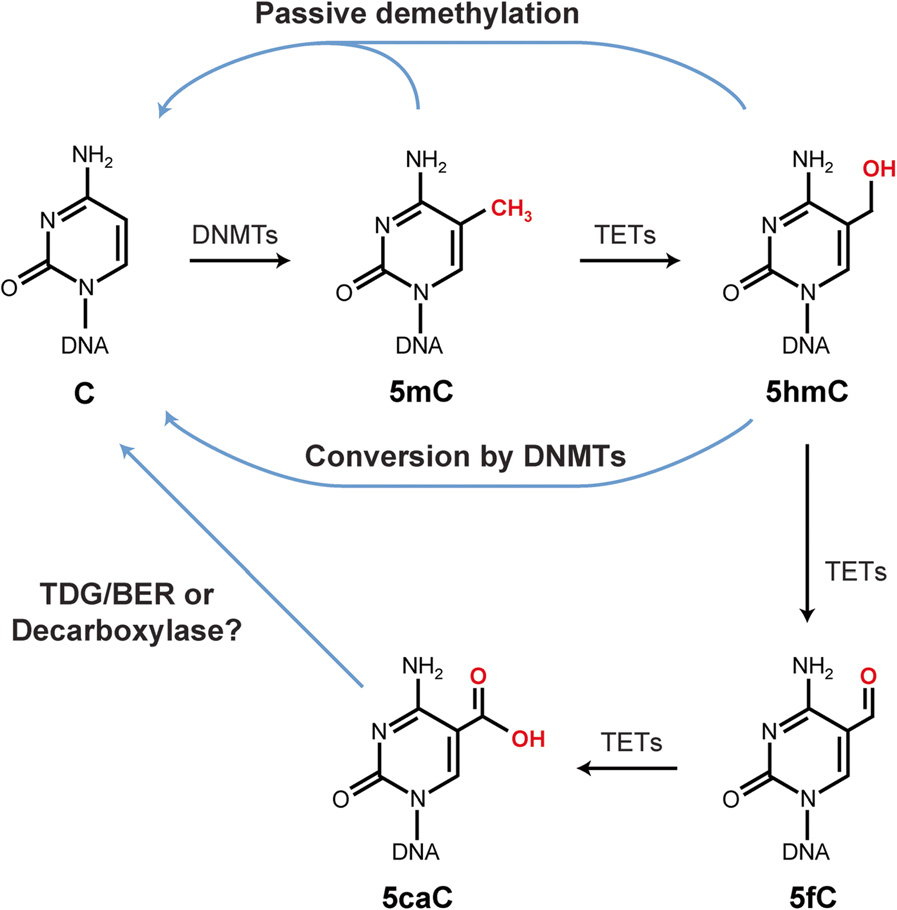
**Chemical structures of 5-methylcytosine (5mC) and its oxidation products 5-hydroxymethylcytosine (5hmC), 5-formylcytosine (5fC) and 5-carboxylcytosine (5caC).** The potential involvement of these modified cytosine bases in several pathways of passive (replication-dependent) and active (replication-independent) DNA demethylation is indicated. One active demethylation pathway is proposed to include consecutive oxidation steps followed by removal of 5caC by thymine DNA glycosylase (TDG) in a base excision repair (BER) scheme or by decarboxylation arriving back at cytosine (C). DNMT, DNA methyltransferase.

We now know that 5hmC levels vary substantially between different cell types and tissues and are highest in the brain, in particular in neurons [6,7,10-12]. Because 5hmC is an oxidation product of 5mC, it is clear that formation of 5hmC from 5mC automatically lowers the levels of 5mC at any given nucleotide position or even genome-wide. Therefore, it was immediately apparent that the conversion of 5mC into 5hmC could be the first step in a pathway leading towards DNA demethylation. There is evidence from various experimental systems that this may indeed be the case [13,14]. The end result of this demethylation pathway is passive or active removal of the modified base and/or disappearance of the methyl group from cytosine in DNA (Figure 1). In the passive demethylation pathway, 5hmC cannot be copied by the maintenance DNA methyltransferase, DNMT1, an enzyme that propagates pre-existing methylation patterns and operates on hemimethylated CpG sites [15,16]. The active demethylation process that uses 5hmC as an intermediate is considerably more complicated. One report suggested that 5hmC can be converted to cytosine by DNA methyltransferases [17]. Deamination of 5hmC produces 5-hydroxymethyluracil [18], which can be removed by base excision repair enzymes including thymine DNA glycosylase (TDG) [19,20] and single-strand selective monofunctional uracil DNA glycosylase (SMUG1) [21]. However, how effectively such a pathway is operative *in vivo* is currently unknown. Stepwise oxidation of 5hmC by TET proteins produces 5-formylcytosine (5fC) and then 5-carboxylcytosine (5caC) [22,23]. This 5caC, which is detectable at low levels in DNA, can then be removed either by base excision repair catalyzed by the DNA glycosylase activity of the protein TDG [23], or by decarboxylation. Theoretically, the decarboxylation pathway should be favorable as it does not require breakage of the DNA phosphodiester bonds, which occurs during TDG-initiated base excision repair. However, to date, no enzymatic activity for the decarboxylation step has been identified although decarboxylation does seem to occur [24].

Many tissues accumulate quite substantial levels of 5hmC, much greater than would be expected if this base were simply a transient intermediate in a sequential oxidation pathway leading towards DNA demethylation. Therefore, 5hmC may be an epigenetic module that has its own unique biochemical coding properties. This function may be a negative or repulsive one since oxidation of the methyl group during production of 5hmC will block binding of proteins that would otherwise interact with 5mC [25]. Alternatively, its function may be a positive or instructive one if proteins exist that specifically bind to 5hmC. So far, several different proteins have shown ability to recognize 5hmC, at least *in vitro*, including UHRF1 [26], MBD3 [27], MeCP2 [28], and several others identified by a proteomics approach [29]. However, the biological role of their binding to 5hmC is still not entirely clear. Most of these proteins have other functions as well, and therefore may not be uniquely designed to interact with 5hmC.

### The role of 5-hydroxymethylcytosine in mammalian development and differentiation

The functional role of 5hmC in mammalian genomes is still unclear. At the beginning of the mammalian life cycle, upon fertilization of oocytes by sperm, most of the 5mC in the paternal (sperm-derived) genome becomes oxidized to form 5hmC [30,31]. This oxidation step, which previously had been thought to reflect true DNA ‘demethylation’ [32,33], is specific to the paternal genome whereas the maternal (oocyte-derived) genome remains protected from Tet-catalyzed oxidation [34,35]. Paternal genome oxidation is catalyzed by Tet3, encoded by the only *Tet* gene expressed at substantial levels in oocytes and zygotes [30]. Genetic knockout of *Tet3* in mice results in failed paternal genome oxidation, compromised development and perinatal lethality [36].

Another important developmental transition involves global DNA demethylation in primordial germ cells (PGCs) that commences at around embryonic day 8.5 to 9.5 and is completed near embryonic day 13.5. The mechanisms of methylation erasure in PGCs have remained largely unclear and controversial. It has long been assumed that replication-independent active DNA demethylation is a key pathway likely involved in this step [37,38]. However, more recent data favor a passive loss of methylation caused by lack of methylation maintenance during DNA replication [39-41]. This passive loss of 5mC may be effectively initiated by conversion of 5mC into 5hmC [42]. Tet1 and Tet2 are the 5mC oxidases most highly expressed in PGCs at this stage [36,43]. Progeny of mice deficient in Tet1 and Tet2 have deficiencies in DNA demethylation at imprinted genes [44]. However, Tet1/2-deficient animals of both sexes were fertile, with females having smaller ovaries and reduced fertility. Deletion of Tet1 and Tet2 can produce viable adults, although the majority of such mice die during embryogenesis or around birth and show various developmental defects [44]. The data suggest that Tet1/2-induced 5mC oxidation in PGCs is not absolutely required for producing viable offspring. The currently available information on DNA demethylation in zygotes and in PGCs still lacks a more specific analysis of 5hmC at the DNA sequence level, as can be accomplished, for example, by TAB-sequencing [45]. It is expected that such information will clarify the global or locus-specific involvement of 5hmC formation in the initiation of passive (or active) DNA demethylation. The previous implication of base excision repair processes in germ line reprogramming [43,46], which by itself would pose a tremendous risk for maintenance of genome integrity if operative at a global level, may have various other explanations. In one scenario, the occurrence of base excision repair activity might be explained by the requirement to counteract spurious non-targeted oxidation reactions catalyzed by Tet oxidase activity on guanines at methylated CpG sites (guanine being the DNA base most susceptible to oxidation). In another setting, 5hmC may be oxidized further, perhaps at specific sequences, by Tet proteins to form 5caC, which is then removed by base excision repair initiated by TDG [23].

Because 5hmC is most abundant in brain tissue, it has become a priority to understand the function of this modified base in the brain. For example, in DNA from human brain cortex, the level of 5hmC is about 1% of all cytosines or 20 to 25% of all 5mC bases [47]. This corresponds to approximately 6,000,000 5hmC bases per haploid genome. Clearly these levels suggest that 5hmC has an important functional role in the mammalian brain. Studies reported so far have shown that 5hmC in brain tissues is very abundant within gene regions, either at promoters or even more so within intragenic regions, the so-called gene bodies [10,12,48]. It is conceivable that the formation of 5hmC at promoters, CpG islands or CpG island shores (edges) functions analogous to a repair process to oxidize and eventually remove inappropriately introduced 5mCs in these regions [10,49]. Deposition of 5hmC in promoters [50] or gene bodies [10,12,51] often correlates positively with gene activity. The mechanism of how gene body-associated 5hmC increases transcript levels is currently unknown. One possibility is that 5mC oxidation releases a repressive effect on transcription, perhaps by counteracting spurious intragenic anti-sense transcription. Other explanations may include the fact that 5hmC has a destabilizing effect on DNA structure [52,53] that potentially favors the opening of the double helix by the transcription apparatus.

5hmC, although not recognized by several methyl-CpG binding proteins, including MBD1, MBD2 and MBD4 [25], is able to bind MeCP2 [28], a methyl-CpG-binding protein that is abundant in the brain and is mutated in the neurological disorder Rett syndrome [54]. Earlier studies, using the methyl-CpG binding domain (MBD) of MeCP2 rather than the full-length protein, did not conclude that MeCP2 binds to 5hmC [55]. The reasons for these discrepancies are not clear. The connection between MeCP2 and 5hmC in the brain is of particular interest since levels of 5hmC are highest in the brain and MeCP2 is an abundant protein in the brain reaching levels similar to that of histone H1. For these reasons, a genome-wide rather than sequence-specific mechanistic role of 5hmC-binding by MeCP2 can be anticipated in the brain.

As shown recently, formation of 5hmC is critical for brain development. The base is abundant in developing neurons in which its level increases relative to neural progenitor cells and where it specifically localizes to gene bodies of genes important for neuronal differentiation [56]. *Tet3* is most highly expressed in the developing mouse brain cortex followed by *Tet2* and the levels of *Tet1* are very low in this tissue. An increase in the levels of *Tet2*, *Tet3* and 5hmC in differentiating neurons coincides with reduction of the Polycomb H3K27 methyltransferase Ezh2 and loss of H3K27me3 at critical genes. Reducing the levels of *Tet2* and *Tet3* or increasing *Ezh2* expression results in incomplete or blocked neuronal differentiation [56]. Thus, formation of 5hmC promotes neuronal differentiation by modulating the expression of genes most critical in this important developmental transition.

### Loss of 5-hydroxymethylcytosine in cancer

The levels of 5hmC in cancer are strongly reduced relative to the corresponding normal tissue surrounding the tumor [47]. Using liquid chromatography-mass spectrometry, anti-5hmC antibody-based immuno-dot blots and immunohistochemistry, we demonstrated tumor-associated loss of 5hmC for cancers of the lung, brain, breast, liver, kidney, prostate, intestine, uterus and melanoma [47]. Other investigators confirmed this observation by showing loss of 5hmC in different types of solid tumors [57-60]. Moreover, reintroduction of TET2 has been shown to restore 5hmC levels and decrease metastatic potential of melanoma cells [61]. Strikingly, when we co-immuno-stained tissue sections with antibodies against 5hmC and against the Ki67 antigen, which is a marker found only in proliferating cells, we observed that 5hmC and Ki67 are almost never present simultaneously in a single cell [47]. At a clinical diagnostic level, combined immunohistochemical analysis of 5hmC loss and presence of Ki67-positive cells could be developed into a biomarker for cancer diagnosis. The lack of, or strong reduction of, 5hmC in tumors suggests that proliferating cells lose 5hmC. In most cases, the bulk tumor mass is depleted of 5hmC even when Ki67-positive cells are rare, suggesting that these tumor cells have had a past history of proliferation leading to loss of 5hmC, which is then not re-established [47]. The replication-dependent loss of 5hmC reflects a situation reminiscent of the one in preimplantation embryos in which initial formation of 5hmC in the paternal DNA is followed by replication-dependent loss or dilution of this mark [30,62]. Similarly, global 5hmC content decreases rapidly as cells from normal tissue adapt to cell culture [51]. The simplest explanation is that oxidation of 5mC produces a hemi-hydroxymethylated CpG site in DNA that is not recognized by DNMT1 during DNA replication. Such an explanation is consistent with *in vitro* studies showing that DNMT1 is unable to operate on CpG sites that contain 5hmC [15,16]. However, other explanations for the reduction of 5hmC in cancer are also possible. The levels of TET proteins may be lower in tumor tissue than in its matching normal tissue counterpart. Although we did not observe consistent differences at the RNA level for *TET1*, *TET2*, or *TET3* in lung and brain tumors relative to normal tissue [47], others have reported lower levels of *TET* gene expression in cancer [58,60]. An additional possibility is that cancer cells contain compromised metabolic pathways that are involved in production of the co-factor for TET activity, 2-oxoglutarate (see below).

### Mutation of *TET2* in human cancer

TET1 belongs to a family of proteins characterized as promoting the conversion of 5mC to 5hmC in mammalian DNA [8,63]. There are three identified family members belonging to the TET family: TET1, TET2, and TET3. TET1 is located on human chromosome 10q21.3, while TET2 is located on chromosome 4q24 and TET3 is on chromosome 2p13.1. The TET1 enzyme consists of a zinc finger CXXC DNA binding domain, a cysteine-rich region, and a 2-oxoglutarate- and iron (II)-dependent dioxygenase (2OGFeDO) domain [8,64]. TET3 also contains an N-terminal CXXC domain [65]. However, the *TET2* gene underwent a chromosomal gene inversion during evolution, thus separating its CXXC domain from the catalytic domain and creating a new CXXC domain gene named *IDAX*/*CXXC4*, which encodes a negative regulator of TET2 [66]. Based on EST profiles and expression arrays, *TET1* shows greatest expression during embryogenesis and does not show relevant expression in adult tissues. *TET2* is mostly expressed in hematopoietic cells and *TET3* seems ubiquitously expressed in adult human tissues.

Leukemia is a disease where, during normal hematopoietic stem cell differentiation, clonal expansion of hematopoietic precursor cells in the bone marrow is affected at a certain stage of differentiation, causing an imbalance between differentiation and self-renewal. Inappropriate expansion of hematopoietic progenitor cells is primarily caused by a blockage of cell maturation. Myelodysplastic syndrome (MDS) disorders in hematopoiesis are characterized by cytopenia (low blood cell count), ineffective hematopoiesis in one cell lineage or another, and an increased risk of transformation to acute myeloid leukemia (AML) [67]. In AML, rapid growth of abnormal white blood cells in the bone marrow leads to a blockage in the production of various cells from other cell lineages.

*TET2* has been found mutated in patients with myeloproliferative neoplasms (MPN), MDS, AML and chronic myelomonocytic leukemia (CMML), and is the most commonly mutated gene in MDS [68-72]. Mutations of *TET1* or *TET3* are not observed in MDS nor does the *TET2* mutation correlate with several other known common mutations [68]. Interestingly, isocitrate dehydrogenase 1/2 (*IDH1/2*) mutations are rarely found together with *TET2* mutations, but have similar effects as *TET2* mutations on hematopoietic stem cells (HSCs) [73]. While *TET2* mutations are associated with reduced overall survival in AML compared to patients with wild-type *TET2*, *TET2* mutations in MDS and MPN patients promote progression to AML [68]. The *TET2* gene is comprised of a total of eleven exons, translating into a 2002 amino acid protein product [70]. *TET2* mutations in myeloid cancers have been most commonly observed within exons 3a and 10, which are the longest exons [71]. Both multipotent and committed progenitor cells in the hematopoietic lineage are targeted by *TET2* mutations in MPN, implying that TET2 plays an important role in myelopoiesis [69]. Deletions of *TET2* and loss of heterozygosity or uni-parental disomy was observed in (9%) MDS/AML patients with mutated *TET2*[70], where it is likely for the wild-type allele to be lost during recombination, allowing mutated *TET2* to promote a loss of function phenotype. Kosmider *et al*. [70] observed that 50% of patients with mutated *TET2* had genetic defects that targeted the two *TET2* copies. Mutations in *TET2* seem to lead to loss of function, suggesting that it may play a tumor suppressive role.

Understanding the underlying implications of mutant TET2 lacking function and its role in myeloid malignancies is a current research priority. Several laboratories generated conditional *Tet2* knockout mouse models [74-77] in which critical *Tet2* exons were targeted. Moran-Crusio *et al*. [74] observed that *Tet*2^−/−^ mice developed splenomegaly at 20 weeks of age, showing phenotypes similar to those observed in human CMML patients with mutant *TET2*. The data from the different mouse models led to similar observations. Deleting *Tet2* is not embryonic lethal. A major observation made by Moran-Crusio *et al*. [74] and by Ko *et al*. [77] is that hematopoietic stem cells from *Tet2*^−/−^ mice have an increased ability to repopulate the hematopoietic compartment *in vivo* during competitive reconstitution assays with competition from HSCs from Tet2^+/+^ cells. Analysis of various organs of *Tet2*^−/−^ mice showed that loss of *Tet2* is not compensated by an increase in *Tet1* or *Tet3* expression [75,77]. 5hmC levels are significantly decreased in bone marrow and spleen of *Tet2*^−/−^ mice [75,77]. *Tet2*^−/−^ mice show an increase in HSCs with a slight increase in myeloid progenitors, skewing hematopoiesis towards monocyte/macrophage cell fates [74-77]. It is suggested that an active Tet2 would regulate normal hematopoiesis to assure proper lineage distribution and controlled differentiation of HSCs. Of particular interest is the effect of *TET2* mutations on levels and patterns of 5mC in the genome. However, the current data are far from clear. Whereas one report indicated that *TET2* mutation in AML is associated with a DNA hypermethylation phenotype [73], other data suggested that bone marrow samples from patients with *TET2* mutations have low 5hmC levels and DNA hypomethylation [78,79]. The situation is complicated by the fact that hematopoietic malignancies are often characterized by mutations in several epigenetic modifiers including *EZH2*, *IDH1*, *IDH2*, *MLL*, *DNMT3A*, and *ASXL1*, thus potentially obscuring any straightforward associations [80]. For example, in one study, eight of eleven patients with *DNMT3A* mutations (73%) in T-cell lymphoma also had *TET2* mutations [81].

### Mutations in co-factor pathways

**Figure 2:**
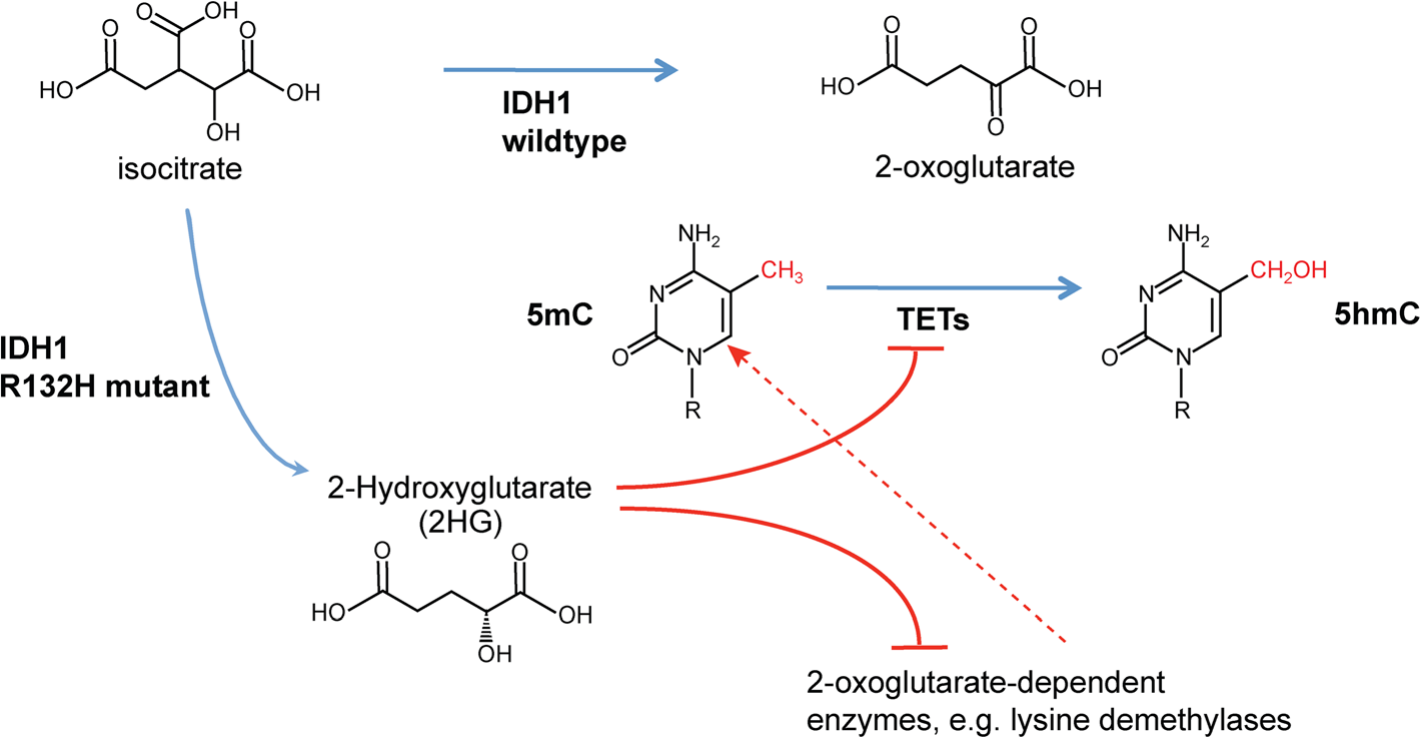
**Production of 2-oxoglutarate by isocitrate dehydrogenase.** 2-oxoglutarate is a cofactor for Ten-eleven translocation (TET) proteins, which oxidize 5-methylcytosine (5mC) to 5-hydroxymethylcytosine (5hmC). The isocitrate dehydrogenase (IDH)1 mutant R132H produces 2-hydroxyglutarate (2HG), a competitive inhibitor of 2-oxoglutarate-dependent enzymes including TET proteins. The inhibition of TET activity or of other 2-oxoglutarate-dependent enzymes by 2HG may affect patterns of 5mC in the genome of IDH1 mutant cells.

5mC oxidases are 2-oxoglutarate-dependent enzymes (Figure 2). This cofactor is produced in the tricarboxylic acid cycle from isocitrate by the enzyme IDH. Interestingly, several types of human tumors contain mutations in the *IDH1* gene. *IDH1* mutations are particularly frequent in grade II and III gliomas where they are found in up to 70% of the patients [82]. Mutations in *IDH1* and *IDH2* are also seen in myeloid leukemias and a few other malignancies but at a lower frequency [73,83]. These *IDH1* mutations are not scattered throughout the gene but are almost exclusively found at amino acid position 132. This finding suggests that this particular IDH1 mutant protein has a gain of function property. A surprising discovery was that the IDH1 codon 132 arginine to histidine mutant produces the oncometabolite 2-hydroxyglutarate (2HG) as a reaction product instead of 2-oxoglutarate [84]. It seems that the isocitrate oxidation reaction carried out by this mutant is incomplete and produces 2HG only. Furthermore, 2HG is a competitive inhibitor of many, if not all, 2-oxoglutarate-dependent enzymatic activities. The TET proteins represent one class of such enzymes, and it was shown that 2HG is an inhibitor of TET1 and TET2 [85].

One interesting correlate of having mutated IDH1 in glioma tumors is that the IDH1-mutant tumors are almost always associated with abundant genome-wide changes in DNA methylation as indicated by widespread hypermethylation of CpG islands [86]. This phenotype has been referred to as CpG-island methylator phenotype (or CIMP) [87]. It is tempting to presume that CIMP in IDH1-mutant gliomas is linked to a failure of 5hmC production in these tumors because TET activity is compromised by 2HG. In fact, experimental introduction of an IDH1 mutant construct into human astrocytes led to the emergence of a CIMP-like phenotype [88]. Furthermore, in conditional knock-in mice in which the most common *Idh1* mutant R132H was inserted into the endogenous *Idh1* locus and was expressed in hematopoietic cells, DNA hypermethylation was observed [89]. However, in a direct comparison of 5hmC levels in DNA between IDH1 mutant and IDH1 wild-type gliomas, we did not observe any substantial differences between these two categories of brain tumors [47]. Therefore, one needs to keep in mind that mutant IDH1 and its metabolite product 2HG not only affect TET enzymes but also inhibit many lysine demethylases that depend on 2-oxoglutarate and other 2-oxoglutarate-dependent enzymes. The dysfunction of these lysine demethylases may have a secondary impact on DNA methylation patterns at CpG islands.

## Conclusion

Emerging data suggest that the DNA base 5hmC has functional roles in gene regulation and development. Many details are not yet understood at a sufficient level. Because of the abundance of 5hmC in brain tissue, a specific function of this base in the brain is most plausible. However, 5hmC occurs also in other tissues. Additional studies will be required to examine its distribution in different tissue types to determine if this base has a more general role in gene control. Single base-level analysis of 5hmC is now possible [45] allowing for a more precise mapping of this base modification in the genome. Additional proteins interacting uniquely with 5hmC will need to be identified. A key question is whether such proteins provide a functional readout to 5hmC by linking the base to mechanistic pathways in chromatin biology. The exact role of 5hmC in DNA demethylation needs more evidence to clearly support such a role. One possibility is that localized loss of 5hmC created by local defects in 5mC oxidation could shift the balance of methylated versus unmethylated cytosines within CpG islands towards the hypermethylated state. If this were the case, a mechanistic explanation for the widespread cancer-associated DNA hypermethylation would be at hand. However, current data suggest that the loss of 5hmC in cancer occurs at a more global scale. How the genome-wide loss of this base could be tied, directly or indirectly, to the prevalent phenomenon of CpG-island hypermethylation in cancer will be an interesting aspect of future studies. At a more practical level, the loss of 5hmC as observed in tumor cells could be used in diagnostic procedures to identify early-stage malignant disease.

## Abbreviations

2HG: 2-hydroxyglutarate; 5caC: 5-carboxylcytosine; 5fC: 5-formylcytosine; 5hmC: 5-hydroxymethylcytosine; 5mC: 5-methylcytosine; AML: acute myeloid leukemia; CIMP: CpG-island methylator phenotype; CMML: chronic myelomonocytic leukemia; DNMT: DNA methyltransferase; HSC: hematopoietic stem cell; IDH: isocitrate dehydrogenase; MBD: methyl-CpG binding domain; MDS: myelodysplastic syndrome; MPN: myeloproliferative neoplasms; PGC: primordial germ cell; SMUG1: single-strand selective monofunctional uracil DNA glycosylase; TDG: thymine DNA glycosylase; TET: Ten-eleven translocation.

## Competing interests

The authors declare that they have no competing interests.

## Authors’ contribution

GPP wrote the manuscript draft and final version of the manuscript. SK and SGJ helped to draft the manuscript. All authors read and approved the final manuscript.
